# The Hydration Characteristics, Structural Properties and Volatile Profile of Squid (*Symplectoteuthis oualaniensis*) Mantle Muscle: Impacts of Steaming, Boiling, and Sous Vide Cooking

**DOI:** 10.3390/foods10071646

**Published:** 2021-07-16

**Authors:** Hong Xiao, Nannan Li, Longtao Yan, Yong Xue

**Affiliations:** 1 Department of Food Science and Engineering, Ocean University of China, Qingdao 266003, China; 11200732001@stu.ouc.edu.cn; 2 Sanya Ocean Institute, Ocean University of China, Sanya 572024, China; 21200731115@stu.ouc.edu.cn (N.L.); 21200731098@stu.ouc.edu.cn (L.Y.)

**Keywords:** hydration characteristics, squid (*Symplectoteuthis oualaniensis*), sous vide, structural changes, volatile profile

## Abstract

Herein, the effects of boiling (BO), steaming (ST), and sous vide (SV) on the hydration characteristics, structural properties, and volatile profile of squid (*Symplectoteuthis oualaniensis*) mantle muscle (SMM) were investigated. Three cooking methods resulted in a dramatic decrease in proton mobility and freedom of protons, the relaxation time T_2_ decreased after cooking, and the water binding in the SMM was closer, but the SV treatment could retain more water in the SMM. SV resulted in a lower cooking loss (10.8%) than ST (49.0%) and BO (36.7%). Samples treated with SV had a better color and texture, the secondary structure β-fold of the squid protein was damaged by cooking to a certain extent, and the damage degree was BO > ST > SV. Compared with BO and ST, SV treatment caused more damage to the myosin heavy chain, paramyosin, and actin in SMM, improved the tenderness of SMM, and resulted in more regular internal reticular structures and less formation of fibrous structures. Cooking methods can significantly affect the volatile components of SMM, resulting in increasing volatile components or generating new volatile components in SMM including 2-methylbutanal, ethyl 2-methylpropanoate, acetic acid, and propyl methyl ketone in ST and BO samples and 2-methylbutanal, hexanal, and 2,3-pentanedione in SV samples. Therefore, SV resulted in the best quality squids and has substantial industrial application potential.

## 1. Introduction

Squid (*Symplectoteuthis oualaniensis*) is widely distributed in equatorial and subtropical waters of the Indian Ocean and Pacific Ocean, squid are abundant in the South China Sea and the northwestern Indian Ocean, and it is estimated that the annual available catch of squid is 1,470,000 to 1,950,000 tons [1]. Moreover, squid muscle is rich in nutrition and contains compounds including protein (17.24%), unsaturated fatty acids (4.34%), amino acids (14.05%), minerals, among others [2]. In addition, squid has gradually become an important aquatic protein source because it is appetizing, nourishing, inexpensive, and has a high fishing amount. However, squid meat is tough and hard to splinter due to the high content of insoluble myostromin (11.0%) [3], and this negatively influences consumer acceptance. At present, most of the squid products on market are dry products, which has hindered the development and higher value application of squid. Accordingly, it is critical to find a reasonable processing method to process and utilize squid.

Boiling water cooking and steam cooking are traditional high temperature processing methods and are used for processing a variety of foods, including vegetables, meats, and seafood products. However, high temperature processing produces a series of chemical and physical reactions such as protein denaturation and lipid oxidation reactions and leads to loss of water-soluble components, vitamins, and minerals [4,5]. Sous vide is a recently emerging cooking method where raw meat is cooked in vacuum-sealed heat-stable bags at low temperatures ranging from 50 °C to 85 °C for prolonged times of up to 48 h. The use of vacuum-sealed bags allows efficient and uniform transfer of heat inside the meat and ensures provision of a safe product by eliminating microbial contamination risk during post-cooking handling and slicing storage. At present, sous vide cooking has become very popular in food processing and has been widely used in food enterprises. Additionally, many studies have reported the application of sous vide cooking in the processing of a variety of meat products, including marinated chicken breast [6], chicken sausage [7], lamb loins [8], Argentine beef [9], pork loin [10], and pork face [11]. Moreover, it was found that, compared with traditional high-temperature processing, sous vide cooking could retain more minerals in meat, improve sensory quality, increase yield and water content [12], reduce lipid oxidation [13,14,15], promote the release of cathepsin L and cathepsin B in lysosomes, and reduce the breaking force of meat [16]. Christensen et al. [17] reported that the tenderization achieved through sous vide cooking is mainly attributed to the reduced denaturation of proteins at typically lower temperatures used, weakening of connective tissue through collagen solubilization, and retention of water, thereby improving the tenderness of meat. In recent years, a series of studies have explored seafood processing with the sous vide method [18,19,20,21]. However, there are no studies on the sous vide processing methods of squid (*Symplectoteuthis oualaniensis*). Thus, exploring sous vide methods for improving the quality of squid (*Symplectoteuthis oualaniensis*) mantle muscle is necessary.

Water accounts for about 80.50% of the total mass of squid (*Symplectoteuthis oualaniensis*) [2] and significantly influences texture characteristics and cooking quality. Meanwhile, different processing methods affect the physicochemical properties of food. However, it is unclear how different cooking treatments affect the hydration characteristics and physicochemical properties of squid (*Symplectoteuthis oualaniensis*). Different cooking methods result in different volatile characteristics. Volatile components are important features of foods that influence consumer preferences. The volatile changes of boiled squid (*Todarodes pasificaus* (*Surume ika*), *Loligo bleekeri* (*Yari ika*)) and the volatile components of squid (*Illex argentinus*) have been investigated [22,23], and the factors affecting flavors were dependent on species of squid [24]. However, no studies were devoted to assessing how the cooking treatments affect the volatile compounds of squid (*Symplectoteuthis oualaniensis*).

Thus, the purpose of this study was to investigate the effect of three kinds of thermal cooking methods (ST, BO, and SV) on the hydration characteristics of squid (*Symplectoteuthis oualaniensis*) mantle muscle (SMM) by the LF-NMR and MRI technique. Meanwhile, the physicochemical properties including color parameters and texture in SMM treated with different cooking methods were also evaluated. In addition, the structure changes and volatile profile of SMM were analyzed. 

## 2. Materials and Methods

### 2.1. Squid Sample Preparation

Ninety-six fresh squid (*Symplectoteuthis oualaniensis*) with an average weight of 65 ± 5 g and body length of 20 ± 2 cm were commercially obtained from Luo Niushan Agricultural Products Processing Industrial Park (Hainan, Hainan province, China) and used in our study; the squid was kept frozen at −18 °C during delivery to the laboratory within 1 day. After being thawed, the skin, head, pen, viscera, and tentacles of squid were removed, and the SMM had a body length of 11–12 cm and a weight of 29.14 ± 2.87 g. The SMM samples were randomly divided into four groups: raw squid (RAW), steaming (ST), boiling (BO), and sous vide (SV) groups. The ST group SMM samples were steamed for 5 min in a steamer above boiling water. The BO group SMM samples were directly dropped in boiling tap water and cooked for 5 min. The SV samples were sous vide packaged in polyethylene pouches, immersed in a water bath, and cooked with sous vide at 50 °C for 60 min. 

### 2.2. Low-Field Nuclear Magnetic Resonance and Magnetic Resonance Imaging Analysis

Low-field nuclear magnetic resonance (LF-NMR) analysis was determined according to previously published methods by Zhang et al. [25] with minor modification. SMM samples of the same parts (approximately 1 cm × 1 cm × 0.5 cm) were taken and measured using NMR instrument. The T_2_ was measured using a τ-value of 100 μs. The Carr–Purcell–Meiboom–Gill (CPMG) parameters were as follows: SW (KHz) was 200, SF (MHz) was 22, RFD (ms) was 0.080, O1 (Hz) was 971,212.7, TW (ms) was 1000, P1 (μs) was 11.00, GR1 (db) was 20, DR was 1, NS was 8, P2 (μs) was 23.00, TD was 1,162,818, and NECH was 18,000. 

Magnetic resonance imaging (MRI) analysis was performed as described by Sun et al. [26] with minor modification; the MRI images of samples were acquired on a Niumag Benchtop Pulsed NMR Analyzer PQ001 too. T_1_ weighted images was recorded using spin-echo imaging sequence with field of view (FOV) of 100 mm × 100 mm, slice gap (mm) was 1.0, slice width (mm) was 1.0, offset slice (mm) was 26.4, average was 2, phase size was 192, and read size was 256. The echo time (TE) was 20 ms and repetition time (TR) was 500 ms.

### 2.3. Determination of Cooking Loss

Cooking loss, which represents the water holding capacity of SMM, was calculated as follows [13]:

(1) $$\mathrm{Cooking}\;\mathrm{loss}\;=\left(\mathrm{Weight}\;\mathrm{initial}-\mathrm{Weight}\;\mathrm{final}\right)/\mathrm{Weight}\;\mathrm{initial}\;\times 100\%$$

### 2.4. Color Measurements

The surface color of SMM samples treated with different cooking methods was determined using a HunterLab ColorQuest XE (HunterLab, Reston, VA, USA); based on the CIE, L* (lightness), a* (redness), b* (yellowness), and whiteness were used to evaluate the difference in the color change system to evaluate the color change. The average reading was calculated based on values obtained from five locations in each sample. The colorimeter was standardized using a white calibration plate and a light trap. Whiteness was calculated using Equation:

(2) $$\mathrm{Whiteness}=100\;-{\left[\left(100-L^{*2}\right)+a^{*2}+b^{*2}\right]}^{1/2}$$

### 2.5. Texture Profile Analysis

Texture profile analysis (TPA) was performed using a texture analyzer (TMS-Pro, Food Technology, Inc., Sterling, VA, USA) by following the report by Nyaisaba et al. [27] with minor modification. SMM samples of the same parts were taken, the SMM samples were cut into 1 cm × 1 cm × 0.5 cm cubes, compression was applied using a cylindrical probe of 25 mm diameter. The trigger force was set at 0.05 N, and the SMM samples were pressed to 50% of their initial thickness at a constant speed of 144 mm/min; the distance separating the probe and the sample base of texturometer was set at 40 mm. The result of force–time curves for TPA was obtained, which included hardness, cohesiveness, springiness, gumminess, and chewiness.

### 2.6. Sodium Dodecyl-Sulfate Polyacrylamide Gel Electrophoresis Analysis

Sodium dodecyl-sulfate polyacrylamide gel electrophoresis (SDS-PAGE) analysis was performed as reported previously Xu et al. [28] with minor modification; briefly, 5 g of crushed SMM sample was dissolved in 45 mL of 5% SDS, and the homogenizer (IKA T18 digital ULTRA-TURRAX, Staufen, Germany) was used to homogenize at 6500 rpm for 2 min; the sample was extracted with 85 °C water bath for 2 h, followed by centrifugation at 10,000× *g* for 30 min, and the supernatant (total protein) was harvested. An amount of 10 μL of extracted samples with 2.5 mg/mL of protein were separated using electrophoresis with 10% separating gel and 5% stacking gel, followed by staining with 0.12% (*w/v*) Coomassie Brilliant Blue R-250.(Solarbio Science & Technology Co., Ltd. Beijing, China) ColorMixed Protein marker (11 kD–245 kD) PR 1920 was used as a protein marker.

### 2.7. Fourier Transform Infrared Spectroscopy

Fourier transform infrared spectroscopy (FTIR) was measured using previously published methods [29]. Briefly, FTIR analysis was performed using a Nicolet iS50 spectrometer (Thermo Scientific, Shanghai, China) equipped with KBr beam splitter. An amount of 10 mg of freeze-dried SMM sample was mixed with 100 mg KBr of dried at 105 °C. The mixture was grinded in an agate cup to a uniform powder, and the mixture was placed in a mold and pressed into a transparent sheet by holding it for 2–3 min at 10–11 MPa using a hydraulic machine. Spectra were recorded in the mid infrared region, the scanning range was 4000–400 cm^−1^, and the scanning resolution was 4 cm^−1^ with 128 scans, accumulation used the OMNIC software. All spectra were appropriately background subtracted.

### 2.8. Scanning Electron Microscopy

Scanning electron microscopy (SEM) was determined using previously published methods [30] with minor modification; briefly, the SMM samples were cut into pieces of about 5 × 5 × 2 mm in size, and the treated SMM samples were fixed in 0.1% glutaraldehyde buffer solution for 1h and in 0.2% osmium tetroxide fixation solution for 10min. After, samples were washed with phosphate buffer for 3 times (10 min/time), dehydrated with gradient ethanol solution for 10 min each (25%, 50%, 70%, 80%, 90%, and 100%), and finally dehydrated with acetone for 10 min. The dehydrated SMM sample was placed in a 100 mL Centrifuge tube, which was frozen at −80 °C for 12 h and freeze-dried for 24 h. The freeze dried SMM sample was fixed on a bronze stub and sputtered with gold. It was then placed in a JSM-7800F scanning electron microscope (JEOL Ltd., Tokyo, Japan) with a 5 kV acceleration voltage to be observed with the scanning electron microscope (SEM) at 200 times and 1000 times magnification.

### 2.9. GC-IMS Analysis of Squid Volatiles

Volatile components were analyzed using a GC-IMS flavor analyzer (FlavourSpec^®^, Dortmund, Germany). Briefly, 2 g of crushed SMM sample was placed in 20 mL headspace vial and sealed. The GC-IMS measured parameters were as follows: type of column: MXT-5 (15 m × 0.53 mm, 1 μm), analysis time: 20 min, column temperature: 55 °C, carrier gas: and high-purity nitrogen (99.99%). Automatic headspace sampling was used; the sampling volume: 500 μL, incubation time: 15 min, incubation temperature: 50 °C, injection needle temperature: 85°C, and incubation speed was 500 r/min. The programmed flow parameters were as follows: carrier gas: high-purity nitrogen (99.99%), 2 mL/min for 2 min; linear increase to 100 mL/min over 18 min; and total run time of 20 min. To avoid cross-contamination, the syringe was automatically flushed for 30 s with nitrogen gas before each analysis and 5 min after each analysis. The n-ketones C4–C9 (Sinopharm Chemical Reagent Beijing Co., Ltd., Beijing, China) were employed as external references to calculate the retention index (RI) of each volatile compound. Volatile organic components (VOCs) were analyzed by laboratory analytical viewer (LAV) and GC × IMS Library Search (FlavourSpec^®^).

### 2.10. Statistical Analysis

All the experiments were performed in triplicate. Results were analyzed using SPSS statistical analysis software (Version 16.0, SPSS Inc., Chicago, IL, USA). Data are presented in Tables 1–3 as means ± SD. When a significant effect (*p* < 0.05) was detected, the comparative analysis between means was conducted using a one-way ANOVA with Tukey’s test.

## 3. Results and Discussion

### 3.1. Hydration Characteristics during ST, BO, and SV Assessed by LF-NMR 

**Figure 1:**
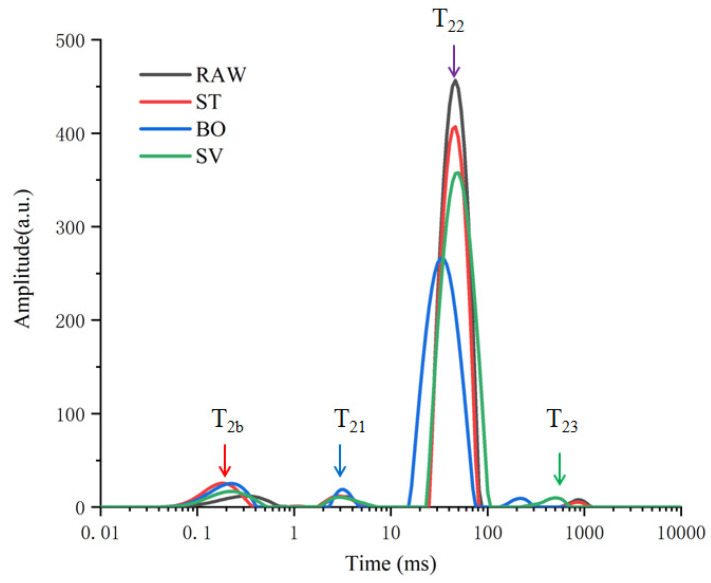
*T*_2_ relaxation time distribution curves of squid (*Symplectoteuthis oualaniensis*) mantle muscle samples before and after treatment of RAW, ST, BO, and SV. RAW: raw squid; ST: steaming; BO: boiling; SV: sous vide.

The physicochemical characters of food can be impacted by thermal food processing by changing protons. The water-binding properties of samples can be determined by LF-NMR. In our study, water distribution and mobility of SMM samples was evaluated by measuring the proton transverse relaxation time (T_2_). The influence of food processing (ST, BO, and SV) on proton was explored, and four peaks were observed in the T_2_ relaxation curves (Figure 1). T_2b_ (<1 ms) represents water that combines with hydrogen bonds, such as carbonyl and amino groups; T_21_ (1–10 ms) represents macromolecules associated water; T_22_ (10–100 ms) represents immobilized water located in myofibrillar network within the space between thin and thick filaments, the main type of water existing in SMM samples; and T_23_ (200–1000 ms) represents low proportion free water on meat surface or that is freely dispersed outside cells [31,32,33]. Both T_2b_ and T_21_ (<10 ms) can be attributed to water of hydration. Therefore, after cooking, free water, immobile water, and bound water are the three main forms of water in the squid. As shown in Figure 1, the T_21_ and T_22_ relaxation time exhibited an evident blue shift from 3.42 to 2.81 ms and from 48.09 to 32.75 ms, respectively, compared with that before cooking. The T_23_ values in ST and BO groups decreased from 870.81 to 229.11 ms, indicating a reduced freedom of protons and proton mobility, but the T_23_ values in SV groups increased to 921.12 ms. During ST and BO processing, a large amount of water in the SMM samples was lost, so the protons were restrained in the cooked meat, resulting in a reduced mobility. 

SMM samples treated with BO exhibited more significant changes compared with those treated with another two types of food-processing method, with T_22_ peak area (A_22_) decreased from 4899.75 to 3697.65 (Table 1), consistent with the findings by Sun et al. [26], who also observed significant changes in the peak area (A_22_) of Spanish mackerel samples during boiling, steaming, roasting, and frying processes. The T_22_ water release caused by three cooking methods was in the following order: SV > ST > BO, and among the three types of food processing methods, BO led to a largest water loss. The T_23_ peak area (A_23_) exhibited an increasing trend after treatment with BO and SV, suggesting that BO and SV treatment induced a slight increase in A_23_ due to the protons expelled from A_22_. The proportion of peak areas of SMM samples before and after treatment with RAW, ST, BO, and SV is shown in Table 1; BO and ST cooking reduced the proportion of peak areas of immobilized water, and the proportion of peak areas of immobilized water decreased from 91.74% to 88.59% and 87.13%, respectively, in the ST and BO group, while P_22_ decreased as the cooking temperature increased, indicating a closer combination between myofibrillar proteins and water. SV cooking increased the proportion of peak areas of free water from 0.87% to 0.96%, indicating that the transformation of free water from the inner increased during SV cooking. Cooking-induced protein crosslink and denaturation would affect water distribution, and the water holding capacity of SMM decreased during high temperature (100 °C) processing.

**Figure 2:**
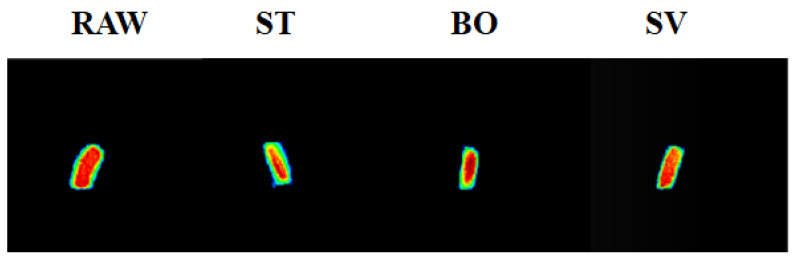
MRI images of the squid (*Symplectoteuthis oualaniensis*) mantle muscle with different cooking treatment. RAW: raw squid; ST: steaming; BO: boiling; SV: sous vide. The blue color indicates a lower proton density, while red color indicates a higher proton density.

MRI has been widely applied in food analysis during food progressing [34]. MRI can be used to determine water distribution in food samples and furthermore to visualize internal structural changes during food processing. Figure 2 shows the MRI images indicating the tomography changes of squid muscle after cooking by ST, BO, and SV. The blue color indicates a lower density, while the red color indicates a higher density. The SMM after ST and BO cooking had a lower intensity compared with RAW samples due to the significant changes of proton state during ST and BO. A similar result was also reported by Xia et al. [35], who also observed the proton changes using MRI with turbot flesh during boiling, stewing, and frying processes. Similar proton state changes were also observed by Sun et al. [26] during steaming and boiling. The decreased intensity of squid muscle after steaming and boiling examined using MRI suggests that both of them are more drastic cooking methods than sous vide for changing the proton state. Our results indicate that compared with ST and BO treatment, SV treatment can retain more moisture in SMM.

### 3.2. Cooking Loss

**Figure 3:**
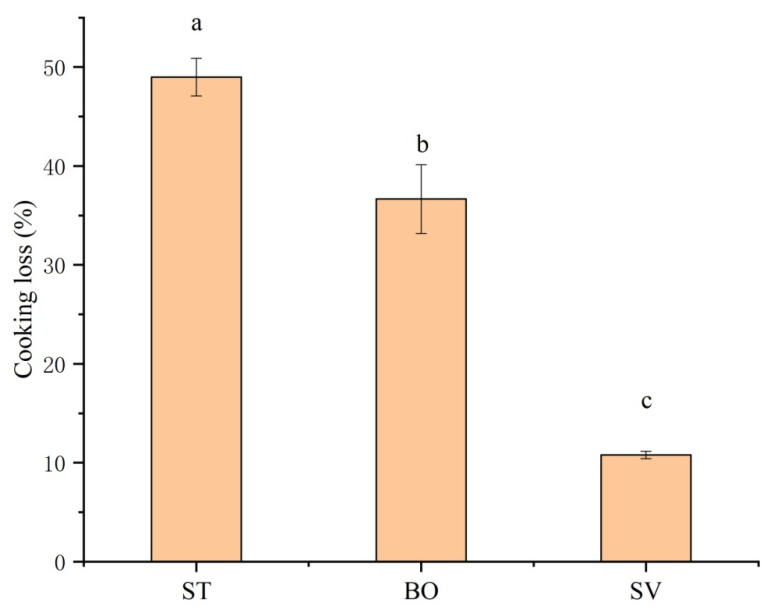
Effect of different cooking methods on cooking loss of squid (*Symplectoteuthis oualaniensis*) mantle muscle. ST: steaming; BO: boiling; SV: sous vide. Different letters indicate significant differences between groups (*p* < 0.05).

Significant cooking loss of SMM including loss of water, peptide, and other flowable small molecules can be induced by thermal food processing. Figure 3 shows that different cooking methods significantly affected cooking loss of SMM (*p* < 0.05), and the cooking loss of SMM samples after ST and BO was 49.0% and 36.7%, respectively, while the cooking loss after SV was only 10.8%. The more significant cooking loss in ST and BO groups compared with that in SV group is because under the SV condition, damage of nutritional compounds and heat-sensitive proteins is minimum [36]. In muscle tissues, myofibrillar proteins capture most of water molecules, which can be lost from the myofibrillar lattice structure with increased cooking temperature due to protein denaturation/degradation or muscle fiber volume reduction [37]. A higher temperature leads to a higher cooking loss of meat. Similar results of cooking loss for steamed Holstein-Friesian bull meat were reported by Modzelewska-Kapituła et al. [38], and similar results for boiled rabbit meat were reported by Rasinska et al. [13]. Our results demonstrate that the SV technique preserved meat juiciness and reduced cooking loss.

### 3.3. Color Analysis

Color can reflect the quality of food products; several factors can induce color change, including Maillard reaction, protein denaturation and oxidation, and generation of different color compounds. Color is highly dependent on food processing [39]. Table 2 shows the color characteristics of SMM samples after ST, BO, and SV. The L* values showed an increase from 61.09 to 68.22, 61.64, and 61.61 for ST, BO, and SV, respectively, indicating that food processing made the SMM samples become brighter than the control, possibly due to protein denaturation and rearrangement, as well as water loss during food processing, and thereby increasing the proportion of light reflection from the squid muscle surface. The a* values showed an increase from 6.16 to 7.95 and 12.53 for ST and BO, respectively, while decreasing to 3.23 in SV groups; the a* values of ST and BO groups but not in SV groups significantly increased due to the protein oxidation and degeneration induced by high temperature processing in ST and BO. The yellowness b*, which reflects the lipid oxidation degree, also showed a similar trend: the b* values showed an increase from 5.01 to 14.64 and 18.35 for ST and BO, respectively, while decreasing to 4.10 in SV groups. Similarly, the high temperature processing in ST and BO also resulted in a larger b* value than that in SV. By contrast, there was no significant difference in the whiteness values between the four groups. 

### 3.4. Texture Profile Analysis

Table 3 shows the changes in texture parameters including hardness, cohesiveness, gumminess, springiness, and chewiness of SMM samples after ST, BO, and SV. Hardness is the magnitude of external force when the food sample reaches a certain deformation [40]; it can be seen that after ST and BO, the hardness increase significantly and that the hardness values showed an increase from 16.60 to 19.15 and 25.69 for ST and BO, respectively, possibly attributed to the protein denaturation and cooking loss. Chewiness refers to the energy required to chew a sample in a stable state [41], and the change of chewiness is positively correlated with hardness; the cohesiveness values increased from 0.37 to 0.63 and 0.66 for ST and BO, respectively. Springiness is the ratio of the height of the second compressed sample to the first one after the sample is deformed [42]; after ST and BO, the springiness of squid muscle increase from 1.31 to 2.09 and 2.21, possibly due to increased production of substances from polymerization during ST and BO. Gumminess represents the property of being cohesive and sticky; after ST and BO treatment, gumminess exhibited a significant increase from 6.09 to 12.13 and 17.09. The chewiness of the ST and BO group was significantly higher than that of raw squid (RAW).

As shown in Table 3, the cohesiveness, hardness, chewiness, gumminess, and springiness of SMM samples in SV group showed a more significant decrease compared with those in other treatment groups. The hardness, springiness, and chewiness of ST and BO were 1.78 and 2.38 times, 1.90 and 1.99 times, 6.45 and 9.52 times of SV, respectively. Meanwhile, we can see that the SMM cooked by ST and BO contracted and curled to varying degrees, while the squid cooked by SV contracted less and did not curl. This phenomenon might be due to the cooking temperature, which is critical for meat tenderization. When the temperature is higher than the collagen shrinkage temperature, it will not decrease the tenderness, while higher temperature will lead to formation of less tender tissues due to the intensive collagen coagulation. Previous studies indicated that beef cooked at 80°C or 90 °C was harder than that cooked at 60 °C or 50 °C [43]. Yin et al. [16] demonstrated that the proteolysis of collagen and myofibrillar protein induced by cathepsin L and cathepsinB, as well as limited longitudinal shrinkage, together contribute to improvement of beef tenderness upon SV.

### 3.5. SDS-PAGE Analysis

**Figure 4:**
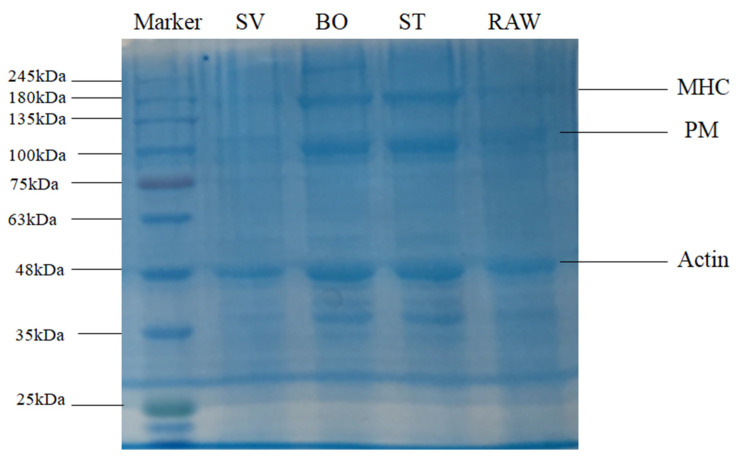
SDS-PAGE patterns of the squid (*Symplectoteuthis oualaniensis*) mantle muscle with different cooking treatment. RAW: raw squid; ST: steaming; BO: boiling; SV: sous vide.

SDS-PAGE was used to examine SMM composition after a variety of treatments. Previous studies showed several electrophoretic protein profiles observed in squid samples corresponding to actin (~45 kDa), paramyosin (PM, ~110 kDa), troponin T (~38 kDa), tropomysin (~40 kDa), and the myosin heavy chain (MHC, ~200 kDa) [27,28], which are particularly common in marine invertebrates [44]. The SDS-PAGE results of SMM samples before and after ST, BO, and SV are shown in Figure 4. Results show the prominent protein profiles of actin, paramyosin (PM), and the myosin heavy chain (MHC) in RAW, ST, and BO treated samples. The intensity of myosin heavy chain (MHC), paramyosin (PM), and actin bands of the SV samples but not of the other treatment samples decreased significantly, consistent with the results reported by Xia et al. [35], who also observed that the intensity of the myosin heavy chain (MHC) band in SV samples remarkably decreased compared with that of the sample cooked at 78 °C, indicating that SV led to MHC hydrolysis. This decrease of band intensity in the SV samples was likely caused by endogenous enzymes. Several previous studies revealed that squid contains endogenous proteolytic enzymes, including cathepsin L, metalloproteinases, cysteine proteinases, and serine protease [27,45,46,47]. Previous studies also demonstrated that endogenous enzymes such as cathepsin B and cathepsin L are involved in MHC, PM, and actin hydrolysis. Ertbjerg et al. [48] found that the existence of highly active cathepsin B and cathepsin L led to more MHC degradation in lactic acid marinated beef. Xia et al. [35] found that the activities of cathepsin L and cathepsin B in SV beef samples were significantly higher than that in the sample cooked under 78 °C. Previous study indicated that proteolysis usually happens under temperature from 25 °C to 75 °C, with the optimum temperature of 40 °C to 50 °C [27]. In the present study, the SMM samples in the SV group were cooked at 50 °C for 60 min. This may cause endogenous proteases to hydrolyze the proteins in SMM. Compared with SV, ST and BO are high temperature heating, and the 100 °C high temperature can inactivate endogenous enzymes; therefore, they cannot hydrolyze the myosin heavy chain (MHC), PM, and actin, and thus the intensity of the protein bands are relatively higher.

### 3.6. Fourier Transform-Infrared (FT-IR) Spectroscopy Analysis

**Figure 5:**
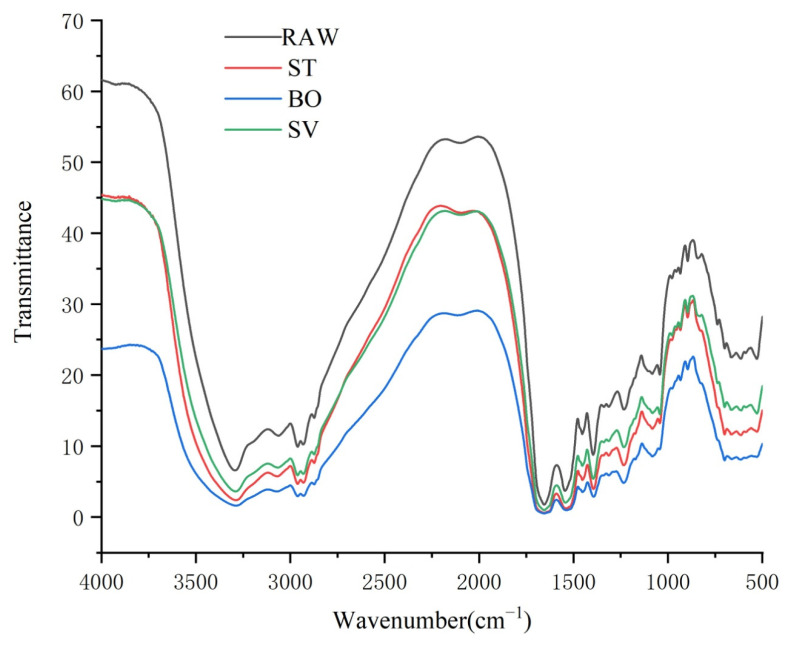
FT-IR spectroscopy of the squid (*symplectoteuthis oualaniensis*) mantle muscle with different cooking treatment. RAW: raw squid; ST: steaming; BO: boiling; SV: sous vide.

FT-IR spectroscopy has been widely used for characterization of protein structural changes, especially in determination of protein secondary structures [49]. Protein secondary structure analysis is generally performed in the amide I and amide II spectral range (1700–1500 cm^−1^). A protein gives rise to many bands in infrared spectroscopy, and among them, the amide I band, resulting from the C = O stretching vibration of the protein backbone, is particularly sensitive to folding patterns in the secondary structural level [50]. Generally, the amide I band consists of overlapped band components falling in 1640–1600 cm^−1^, 1650–1640 cm^−1^, 1660–1650 cm^−1^, and 1695–1660 cm^−1^ ranges, which are attributable to β-sheet, random coil structures, α-helix, and β-turn, respectively [51]. As shown in Figure 5, the peak of SMM protein was close to 1600 cm^−1^, indicating that the secondary structure of SMM protein was primarily β-turn. Moreover, we found that the peak area and height of SMM protein cooked by ST, BO, and SV were both lower than that of RAW, which means that the β-folded structure of protein in SMM was damaged by cooking, and the damage level was BO > ST > SV (Figure 5).

### 3.7. Microstructure of SMM Treated with Different Cooking Methods

**Figure 6:**
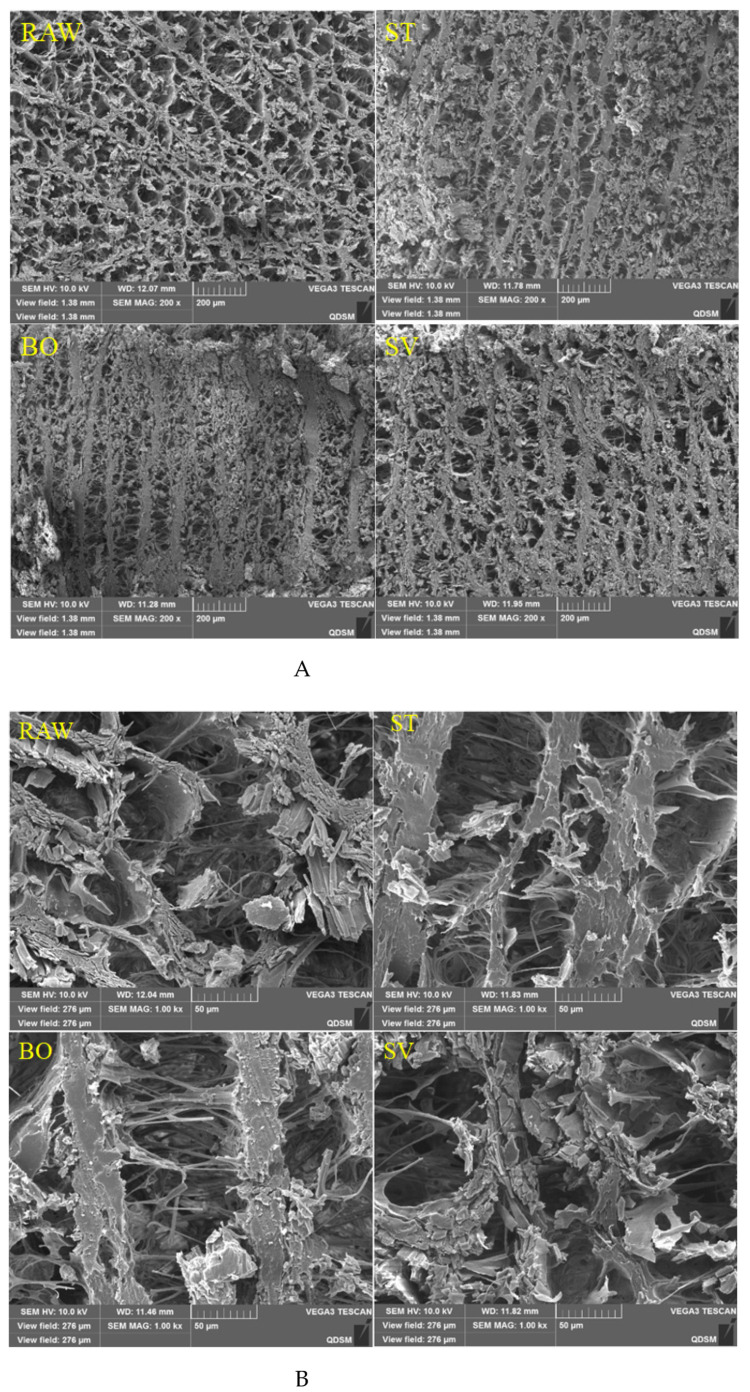
Scanning electron microscopy images of microstructures of squid (*Symplectoteuthis oualaniensis*) mantle muscle before and after treatment of RAW, ST, BO, and SV (**A**: magnification, 200×; **B**: magnification, 1000×;). RAW: raw squid; ST: steaming; BO: boiling; SV: sous vide.

The microstructural changes of SMM treated with different cooking methods were analyzed using SEM. As shown in Figure 6, it was observed that the SMM in the RAW group had an obvious reticular structure formed by intersecting stripes and that the reticular structure was relaxed, regular, and compact. After ST and BO treatment, the SMMs developed “fibrosis” and the reticular structure was destroyed, and there were only obvious vertical stripes. However, after SV treatment, the squid mantle “fibrosis” was not obvious, but there was a certain network structure. This may be due to severe dehydration of SMM caused by ST and BO treatment at high temperatures, resulting in dramatic muscle contraction, and the internal structure of the SMM changed from a network to a vertical streak.

### 3.8. GC-IMS Analysis Results on Squid Volatiles

**Figure 7:**
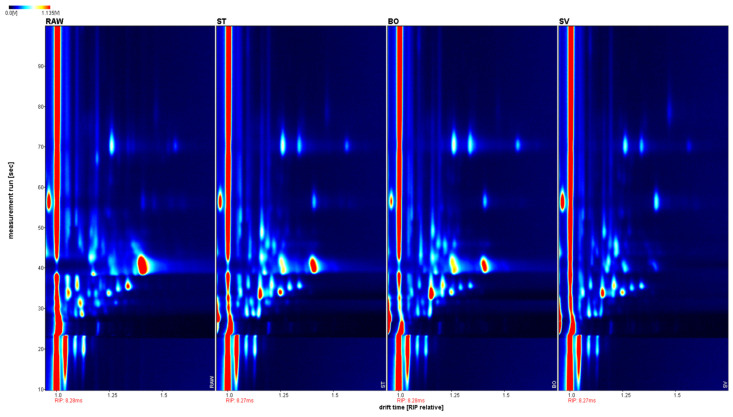
Contrast spectrum of volatile compounds in squid (*Symplectoteuthis oualaniensis*) mantle muscle from different cooking methods. The topographic plot of RAW was selected as a reference, while the plots of other samples were deducted from the reference. Each point on the spectrum represents a volatile component and its color indicates the signal intensity. Red indicates high intensity, and blue indicates low intensity. From left to right, the sample treatments were RAW, ST, BO, and SV, respectively. RAW: raw squid; ST: steaming; BO: boiling; SV: sous vide.

The volatile components of squid (*Symplectoteuthis oualaniensis*) mantle muscle obtained using different cooking methods are shown in Figure 7. The drift time shown was based on the normalization of reactive ion peak (RIP) and ion migration time. The retention time was from 10 to 100 s, and drift time was from 1.0 to 1.8 s. To distinguish the characteristics of volatile components from differently cooked SMM samples, the difference comparison model was applied. The topographic plot of RAW was deducted from the plots of other samples. ST, BO, and SV exhibited obvious red signals, suggesting that the volatile component concentrations in RAW were different from those of ST, BO, and SV. When the sample of ST was used as the reference, RAW and SV exhibited obvious red signals; the topographic plot of BO was close to the reference; therefore, its background was almost white after deduction. Different from RAW and SV, some volatile component concentrations of ST were slightly lower than those in BO, thereby exhibiting a light red background. When the sample of SV was used as the reference, RAW, ST, and BO exhibited obvious red signals, suggesting that the concentrations of volatile components in SV were different from those of RAW, ST, and BO. Steaming and boiling are typical cooking methods for SMM. The high temperature during cooking could increase the production and release of volatile components in SMM. In contrast, SV heats samples for a long time, followed by low temperature under vacuum. Therefore, the volatile components in SV are significantly different from those in BO and ST.

The changes of volatile compounds in SMM treated with different cooking methods were analyzed using GC-IMS separation. As shown in Figure 7, based on the ion migration time and retention time of volatile substances, a total of 38 volatile compounds were detected, and 18 specific volatile compounds were determined in SMM. The following volatile compounds were identified: aldehydes, alcohols, ketones, esters, acid, 3-butenenitrile and hexamethyldisiloxane. The aldehydes detected included pentanal, 3-methylbutanal, 2-methylbutanal, and hexanal; the alcohols detected included 1-butanol and 3-methyl-3-buten-1-ol; the ketones detected included acetoin, propyl methyl ketone, and 2,3-pentanedione; the esters detected included propyl acetate and ethyl 2-methylpropanoate; and the acid detected included acetic acid and propionic acid. Cui et al. [23] analyzed the volatile components of squid (*Illex argentinus*) cooked with different cooking methods, detected 99 volatile compounds, and determined 43 specific volatile compounds. The difference of the results may be related to different detection and analysis methods, different squid varieties, processing temperature, and GC × IMS Library Search retrieval database.

**Figure 8:**
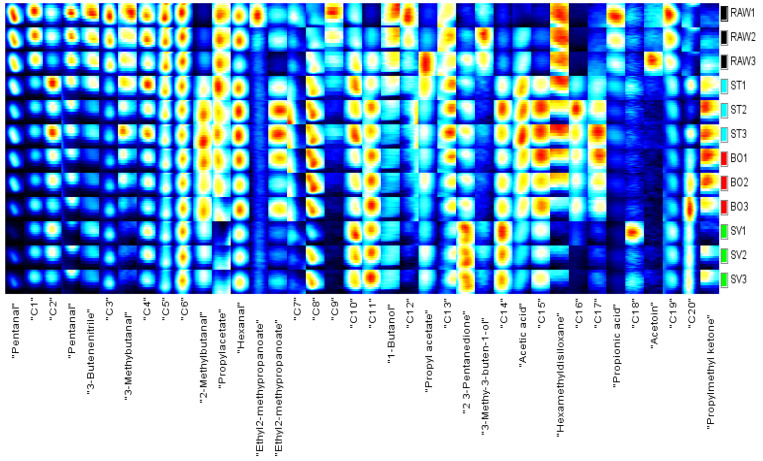
Fingerprint of the squid (*Symplectoteuthis oualaniensis*) mantle muscle with different cooking treatment. RAW: raw squid; ST: steaming; BO: boiling; SV: sous vide. The *y* axis on the right side of the GC-IMS fingerprint is the SMM sample number, and the *x* axis is the name of the volatile compounds; the numbers C1–C20 indicate the compounds that had not been identified.

In order to identify characteristic peak regions of the squid (*Symplectoteuthis oualaniensis*) mantle muscle with different cooking treatment, fingerprint was reconstructed according to all spectral peaks in GC-IMS two-dimensional map (Figure 8). The *y* axis on the right side of the GC-IMS fingerprint is the SMM sample number, and the *x* axis is the name of volatile compounds; the numbers C1–C20 indicate the compounds that had not been identified. As shown in Figure 7, the concentrations of pentanal, 3-butenenitrile, 3-methylbutanal, 1-butanol, 3-methyl-3-buten-1-ol, and propionic acid in the RAW samples were higher than those in the other SMM samples. The concentrations of 2-methylbutanal, ethyl 2-methylpropanoate, acetic acid, and propyl methyl ketone in the ST and BO samples were higher than those in RAW and SV samples. Higher concentrations of 2-methylbutanal, hexanal, 2,3-pentanedione, and propyl methyl ketone were found in the SV samples than in other samples. The concentrations of some volatile components were lowered after cooking, such as pentanal, 3-butenenitrile, propyl acetate, 1-butanol, and propionic acid, while some volatile components were enhanced after cooking, including 2-methylbutanal, ethyl 2-methylpropanoate, 2,3-pentanedione, acetic acid, and propyl methyl ketone. Aldehydes are the main products of oxidative degradation of fatty acids, and aldehydes were present in all three kinds of cooked squid. These aldehydes include hexanal, nonanal, octanal, heptanal, pentanal, 2-hexenal, and benzaldehyde, which are the characteristic aroma components of meat, and the threshold value of aldehydes is relatively low, which significantly contributes to the flavor of cooked squid. Ketones are also products of fatty acid oxidation, and products of automatic oxidation of unsaturated fatty acids including propyl methyl ketone, propyl methyl ketone, and 2,3-pentanedione were detected in ST, BO, and SV samples. Ester compounds, the products of esterification of alcohols and acids, were found in many crustacean fish products cooked and heated. Ethyl 2-methylpropanoate was present in SV cooked squid. The decrease or increase in the concentrations of compounds in SMM might be attributable to the reaction or decomposition induced by thermal treatment. Cui et al. [23] found the highest contents of 1-propene-3-methylthio, furaneol, linalool, and nonanoic acid in raw squid (*Illex argentinus*); heptanal and N, N-diethylethanamine in BO squid; furfural and 2-methyl-1-propanol in ST squid; and n-propyl acetate and acetic acid ethyl ester in SV squid, which are inconsistent with our results, and the possible reason is the difference of squid species. Deng et al. [52] found the highest contents of acetoin, pentadecanoic acid, dimethylamine, furfural, and n-hexadecanoic acid in raw North Pacific squid (*Todarodes pacificus*); benzothiazole, n-hexadecanoic acid, benzaldehyde, benzyl alcohol, and pyridine in freeze-dried squids; acetoin, methional, pyrazine, 2,6-dimethyl, benzyl alcohol, and benzaldehyde in hot-air-dried squids; and acetoin, n-hexadecanoic acid, tetradecane, tetradecanoic acid, and benzothiazole in heat pump-dried squids. Therefore, our results showed that cooking methods can significantly affect the volatile components of squids.

**Figure 9:**
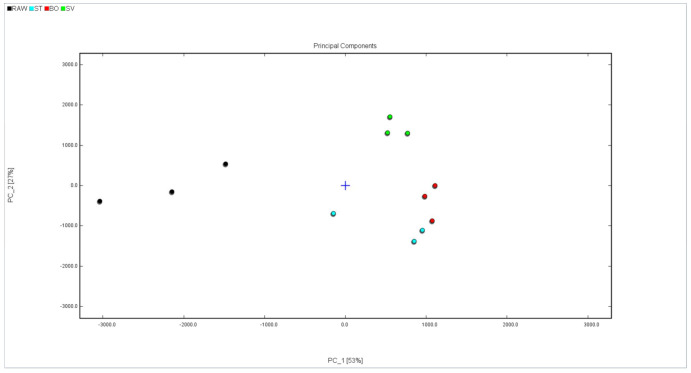
PCA analysis of volatile compounds in squid (*Symplectoteuthis oualaniensis*) mantle muscle from different cooking methods. RAW: raw squid; ST: steaming; BO: boiling; SV: sous vide.

In order to more intuitively analyze the flavor substance differences of the squid (*Symplectoteuthis oualaniensis*) mantle muscle with different cooking treatments, the Dynamic PCA plug-in program was used to conduct a principal component analysis diagram, and the result is shown in Figure 9. The black dots in Figure 8 show the sample of RAW, the blue dots show the sample of SMM cooked with steaming, the red dots show the sample of SMM cooked with boiling, and the green dots show the sample of SMM cooked with sous vide. The cumulative variance contribution rate of PC1 (53%) and PC2 (27%) was 80%, and there was a good separation between the four groups of samples (RAW, ST, BO, and SV), suggesting that the characteristic flavor substances of the samples were different to some extent. The RAW group is clustered on the left side of the PCA diagram, the SV group is clustered on the bottom side of the PCA diagram, and the ST and BO groups are clustered in the middle of the PCA diagram. The main component of the ST group is close to that of the BO group, which may be because high temperature processing has a greater effect on the flavor of SMM. These data suggest that the volatile profile of RAW differed from that of the cooked SMM, further confirming that the volatile profiles of SMM were significantly changed after cooking, consistent with the conclusion obtained from the previous fingerprint.

## 4. Conclusions

In this study, a significantly decreased proton mobility and reduced freedom of protons were observed. LF-NMR and MRI were used to monitor the moisture change during the cooking processes, and results show that the SV technique resulted in a lower water loss, effectively improving the color and increasing textural parameters of SMM. In addition, SV treatment hydrolyzed the myosin heavy chain, paramyosin, and actin in SMM; therefore, it is beneficial to tenderize SMM. Cooking methods can significantly affect the volatile components of SMM. Compared with ST and BO, SV can significantly improve the quality of squid meat, which is an ideal hot processing method for squid meat. Further research is needed to explore the mechanism underlying tenderization of squid meat by SV processing from the perspective of endogenous enzymes. Our findings can be used to improve the quality of squid. This study provides a theoretical reference for the mass industrial production of squid.

## Figures and Tables

**Table 1:** LF-NMR parameters of peak areas (A) of squid (*Symplectoteuthis oualaniensis*) mantle muscle samples before and after treatment of RAW, ST, BO, and SV.

Treatment	A2b	A21	A22	A23
RAW	279.36 ± 42.09 ^a^	11.34 ± 12.06 ^a^	4899.75 ± 158.87 ^a^	46.66 ± 3.51 ^a^
ST	362.41 ± 55.72 ^b^	139.25 ± 13.83 ^b^	4074.15 ± 333.28 ^b^	31. 35 ± 4.64 ^a^
BO	371.65 ± 14.19 ^b^	120.53 ± 1.33 ^a^	3697.65 ± 161.04 ^b^	52.69 ± 22.00 ^a^
SV	248.95 ± 40.67 ^a^	131.36 ± 24.36 ^a^	4507.95 ± 546.77 ^a^	47.95 ± 24.42 ^a^

RAW: raw squid; ST: steaming; BO: boiling; SV: sous vide. Different letters indicate significant differences between groups (*p* < 0.05).

**Table 2:** L*, a*, b* and whiteness values of squid (*Symplectoteuthis oualaniensis*) mantle muscle samples at different cooking methods.

Treatment	L*	a*	b*	Whiteness
RAW	61.09 ± 1.09 ^a^	6.16 ± 2.19 ^b^	5.01 ± 0.99 ^c^	60.24 ± 1.50 ^a^
ST	68.22 ± 4.28 ^a^	7.95 ± 4.17 ^a^	14.64 ± 1.80 ^b^	63.86 ± 4.12 ^a^
BO	61.64 ± 5.27 ^a^	12.53 ± 2.91 ^a^	18.35 ± 1.79 ^a^	55.56 ± 5.36 ^a^
SV	61.61 ± 3.30 ^a^	3.23 ± 0.24 ^b^	4.10 ± 0.51 ^c^	61.26 ± 3.23 ^a^

RAW: raw squid; ST: steaming; BO: boiling; SV: sous vide. Different letters indicate significant differences between groups (*p* < 0.05).

**Table 3:** Texture analysis results of squid (*Symplectoteuthis* *oualaniensis*) mantle muscle samples at different cooking methods.

Treatment	Hardness (N)	Cohesiveness	Springiness	Gumminess	Chewiness
RAW	16.60 ± 1.07 ^b^	0.37 ± 0.04 ^b^	1.31 ± 0.20 ^b^	6.09 ± 0.87 ^c^	8.09 ± 2.21 ^c^
ST	19.15 ± 2.02 ^b^	0.63 ± 0.03 ^a^	2.09 ± 0.31 ^a^	12.13 ± 1.23 ^b^	25.62 ± 5.86 ^b^
BO	25.69 ± 1.45 ^a^	0.66 ± 0.02 ^a^	2.21 ± 0.07 ^a^	17.09 ± 1.32 ^a^	37.78 ± 2.84 ^a^
SV	10.81 ± 2.35 ^c^	0.33 ± 0.03 ^b^	1.11 ± 0.16 ^b^	3.35 ± 0.40 ^d^	3.97 ± 0.84 ^c^

RAW: raw squid; ST: steaming; BO: boiling; SV: sous vide. Different letters indicate significant differences between groups (*p* < 0.05).

## References

[1] Zhang L.N., Zeng S.K., Qiu Y., Lin H.S., Zhang C.H. (2020). Effects of heating temperature and time on the surface microstructure of *Symplectoteuthis oualaniensis*. Fish. Mod..

[2] Yu G., Zhang H.J., Yang S.L., Yang X.Q., Hao S.X., Zhang P., Lin W.L. (2014). Nutritional component analysis and quality evaluation of *Ryukyu squid* in South China sea. Sci. Technol. Food Ind..

[3] Amonrat T., Soottawat B., Wonnop V. (2006). Chemical composition and thermal property of cuttlefish (*Sepia pharaonis*) muscle. J. Food Compos. Anal..

[4] Campo M.M., Muela E., Olleta J.L., Moreno L.A., Santaliestra-Pasías A.M., Mesana M.I., Sañudoa C. (2013). Influence of cooking method on the nutrient composition of Spanish light lamb. J. Food Compos. Anal..

[5] Lopes A.F., Alfaia C.M.M., Partidário A.M.C.P.C., Lemos J.P.C., Prates J.A.M. (2015). Influence of household cooking methods on amino acids and minerals of Barrosã-PDO veal. Meat Sci..

[6] Karyotis D., Skandamis P.N., Juneja V.K. (2017). Thermal inactivation of *Listeria monocytogenes* and *Salmonella* spp. in sous-vide processed marinated chicken breast. Food Res. Int..

[7] Naveena B.M., Khansole P.S., Shashi Kumar M., Krishnaiah N., Kulkarni V.V., Deepak S. (2017). Effect of sous-vide processing on physicochemical, ultrastructural, microbial and sensory changes in vacuum packaged chicken sausages. Food Sci. Technol. Int..

[8] Roldan M., Antequera T., Martin A., Mayoral A.I., Ruiz J. (2013). Effect of different temperature-time combinations on physicochemical, microbiological, textural and structural features of sous-vide cooked lamb loins. Meat Sci..

[9] Grigioni G., Langman L., Szerman N., Iruruet M., Vaudagna S.R. (2008). Effect of whey protein concentrate and sodium chloride concentrations on the odour profle of sous vide cooked whole-muscle beef from Argentina. Meat Sci..

[10] Diaz P., Nieto G., Garrido M.D., Banon S. (2008). Microbial, physical-chemical and sensory spoilage during the refrigerated storage of cooked pork loin processed by the sous vide method. Meat Sci..

[11] Del Pulgar J.S., Gazquez A., Ruiz-Carrascal J. (2012). Physico-chemical, textural and structural characteristics of sous-vide cooked pork cheeks as affected by vacuum, cooking temperature, and cooking time. Meat Sci..

[12] Głuchowski A., Czarniecka-Skubina E., Wasiak-Zys G., Nowak D. (2019). Effect of Various Cooking Methods on Technological and Sensory Quality of Atlantic Salmon (*Salmo salar*). Foods.

[13] Rasinska E., Rutkowska J., Czarniecka-Skubina E., Tambor K. (2019). Effects of cooking methods on changes in fatty acids contents, lipid oxidation and volatile compounds of rabbit meat. LWT Food Sci. Technol..

[14] Cropotovaa J., Mozuraityteb R., Standalb I.B., Rustad T. (2019). Assessment of lipid oxidation in Atlantic mackerel (*Scomber scombrus*) subjected to different antioxidant and sous-vide cooking treatments by conventional and fluorescence microscopy methods. Food Control.

[15] Ortuño J., Mateo L., Rodríguez-Estrada M.T., Bañón S. (2020). Effects of *sous vide* vs grilling methods on lamb meat colour and lipid stability during cooking and heated display. Meat Sci..

[16] Yin Y.T., Pereira J., Zhou L., Lorenzo J.M., Tian X.N., Zhang W.W. (2020). Insight into the Effects of Sous Vide on Cathepsin B and L Activities, Protein Degradation and the Ultrastructure of Beef. Foods.

[17] Christensen L., Ertbjerg P., Løje H., Risbo J., van den Berg F.W.J., Christensen M. (2013). Relationship between meat toughness and properties of connective tissue from cows and young bulls heat treated at low temperatures for prolonged times. Meat Sci..

[18] Espinosa M.C., Díaz P., Linares M.B., Teruel M.R., Garrido M.D. (2015). Quality characteristics of sous vide ready to eat seabream processed by high pressure. LWT Food Sci. Technol..

[19] Martínez-Alvarez O., López-Caballero M.E., Gómez-Guillén M.D.C., Montero P. (2009). The effect of several cooking treatments on subsequent chilled storage of thawed deep water pink shrimp (*Parapenaeus longirostris*) treated with different melanosis-inhibiting formulas. LWT Food Sci. Technol..

[20] Singh C.B., Kumari N., Senapati S.R., Lekshmi M., Nagalakshmi K., Balange A.K., Chouksey M.K., Venkateshwarlu G., Xavier K.M. (2016). Sous vide processed ready-to-cook seerfish steaks: Process optimization by response surface methodology and its quality evaluation. LWT Food Sci. Technol..

[21] Bongiorno T., Tulli F., Comi G., Sensidoni A., Andyanto D., Iacumin L. (2018). Sous vide cook-chill mussel (*Mytilus galloprovincialis*): Evaluation of chemical, microbiological and sensory quality during chilled storage (3 °C). LWT Food Sci. Technol..

[22] Morita K., Kubota K., Aishima T. (2002). Investigating Influence of pH and Parts on Sensory Characteristics and Volatile Components in Boiled Squid Using Experimental Designs. J. Food Sci..

[23] Cui Z.K., Yan H., Manoli T., Mo H., Li H.B., Zhang H. (2020). Changes in the volatile components of squid (illex argentinus) for different cooking methods via headspace–gas chromatography–ion mobility spectrometry. Food Sci. Nutr..

[24] Fratini G., Lois S., Pazos M., Parisi G., Medina I. (2012). Volatile profile of Atlantic shellfish species by HS-SPME GC/MS. Food Res. Int..

[25] Zhang T., Li Z.J., Wang Y.M., Xue Y., Xue C.H. (2016). Effects of konjac glucomannan on heat-induced changes of physicochemical and structural properties of surimi gels. Food Res. Int..

[26] Sun S., Wang S.Q., Lin R., Cheng S.S., Yuan B., Wang Z.X., Tan M.Q. (2020). Effect of Different Cooking Methods on Proton Dynamics and Physicochemical Attributes in Spanish Mackerel Assessed by Low-Field NMR. Foods..

[27] Nyaisaba B.M., Miao W., Hatab S., Siloam A., Chen M., Deng S. (2019). Effects of cold atmospheric plasma on squid proteases and gel properties of protein concentrate from squid (*Argentinus ilex*) mantle. Food Chem..

[28] Xu J.H., Cao H.J., Zhang B., Yao H. (2020). The mechanistic effect of bromelain and papain on tenderization in jumbo squid (*Dosidicus gigas*) muscle. Food Res. Int..

[29] Feng D.D., Xue Y., Li Z.J., Wang Y.M., Xue C.H. (2017). Effects of Microwave Radiation and Water Bath Heating on the Physicochemical Properties of Actomyosin from Silver Carp (*Hypophthalmichthys molitrix*) during Setting. J. Food Process. Preserv..

[30] Cheng S.S., Wang X.H., Yang H.M., Lin R., Wang H.T., Tan M.Q. (2020). Characterization of moisture migration of beef during refrigeration storage by low-field NMR and its relationship to beef quality. J. Sci. Food Agric..

[31] McDonnell C.K., Allen P., Duggan E., Arimi J.M., Casey E., Duane G., Lyng J.G. (2013). The effect of salt and fibre direction on water dynamics, distribution and mobility in pork muscle: A low field NMR study. Meat Sci..

[32] Li C.B., Liu D.Y., Zhou G.H., Xu X.L., Qi J., Shi P.L., Xia T.L. (2012). Meat quality and cooking attributes of thawed pork with different low field NMR T 21. Meat Sci..

[33] Song Y.K., Zang X., Kamal T., Bi J.R., Cong S., Zhu B.W., Tan M.Q. (2018). Real-time detection of water dynamics in abalone (*Haliotis discus hannai* Ino) during drying and rehydration processes assessed by LF-NMR and MRI. Dry Technol..

[34] Aursand I.G., Veliyulin E., Böcker U., Ofstad R., Rustad T., Erikson U. (2008). Water and salt distribution in Atlantic salmon (*Salmo salar*) studied by low-field 1H NMR, 1H and 23Na MRI and light microscopy: Effects of raw material quality and brine salting. J. Agric. Food Chem..

[35] Xia K., Xu W., Huang L., Song Y., Zhu B.W., Tan M.Q. (2018). Water dynamics of turbot flesh during frying, boiling, and stewing processes and its relationship with color and texture properties: Low-field NMR and MRI studies. J. Food Process. Preserv..

[36] Pathare P.B., Roskilly A.P. (2016). Quality and energy evaluation in meat cooking. Food Eng. Rev..

[37] Sikes A.L., Warner R. (2016). Application of high hydrostatic pressure for meat tenderization. Innov. Food Process. Technol..

[38] Modzelewska-Kapituła M., Pietrzak-Fiećko R., Tkacz K., Draszanowska A., Więk A. (2019). Influence of sous vide and steam cooking on mineral contents, fatty acid composition and tenderness of semimembranosus muscle from Holstein Friesian bulls. Meat Sci..

[39] Aroeira C.N., de Almeida Torres Filho R., Fontes P.R., de Lemos Souza Ramos A., de Miranda Gomide L.A., Ladeira M.M., Ramos E.M. (2017). Effect of freezing prior to aging on myoglobin redox forms and CIE color of beef from Nellore and Aberdeen Angus cattle. Meat Sci..

[40] Cai L., Wu X., Li X., Zhong K., Li Y., Li J. (2014). Effects of different freezing treatments on physicochemical responses and microbial characteristics of Japanese sea bass (*Lateolabrax japonicas*) fillets during refrigerated storage. LWT Food Sci. Technol..

[41] Chen T.H., Zhu Y.P., Han M.Y., Wang P., Wei R., Xu X.L., Zhou G.H. (2017). Classification of chicken muscle with different freeze–thaw cycles using impedance and physicochemical properties. J. Food Eng..

[42] Qi J., Li C., Chen Y., Gao F., Xu X., Zhou G. (2012). Changes in meat quality of ovine longissimus dorsi muscle in response to repeated freeze and thaw. Meat Sci..

[43] Palka K. (2003). The influence of post-mortem ageing and roasting on the microstructure, texture and collagen solubility of bovine semitendinosus muscle. Meat Sci..

[44] Geng J.T., Kaido T., Kasukawa M., Zhong C., Sun L.C., Okazaki E., Osako K. (2015). Mechanism study of high browning degree of mantle muscle meat from Japanese common squid Todarodes pacificus during air-drying. Food Chem..

[45] Cardenas-Lopez J.L., Haard N.F. (2005). Cysteine protease activity in Jumbo squid (*Dosidicus gigas*) hepatopancreas extracts. J. Food Biochem..

[46] Cardenas-Lopez J.L., Haard N.F. (2009). Identification of a cysteine proteinase from Jumbo squid (*Dosidicus gigas*) hepatopancreas as cathepsin L. Food Chem..

[47] Tian Y., Umezawa E., Rui D., Konno K. (2010). Three types of proteinase in squid (*Todarodes pacificus*) hepatopancreas as studied by using carp myofibrils as substrate. Fish. Sci..

[48] Ertbjerg P., Mielche M.M., Larsen L.M., Miller A.J. (1999). Relationship between proteolytic changes and tenderness in prerigor lactic acid marinated beef. J. Sci. Food Agric..

[49] Si J.L., Zheng J.Q., Li H., Zhang Y.L. (2015). Effect of salt content on the denaturation of pike eel (Muraenesox cinereus Forsskal, 1775) actomyosin. J. Appl. Ichthyol..

[50] Jackson M., Mantsch H.H. (1992). Halogenated alcohols as solvents for proteins: FTIR spectroscopic studies. Biochim. Biophys. Acta.

[51] Chan J.K., Gill T.A., Paulson A.T. (1992). The dynamics of thermal denaturation of fish myosins. Food Res. Int..

[52] Deng Y., Luo Y.L., Wang Y.G., Zhao Y.Y. (2015). Effect of different drying methods on the myosin structure, amino acid composition, protein digestibility and volatile profile of squid fillets. Food Chem..

